# Blood‐based Alzheimer's disease biomarkers in a behavioral neurology clinic: Real‐world implementation, clinical utility, and diagnostic performance

**DOI:** 10.1002/alz.70891

**Published:** 2025-11-13

**Authors:** Igor Prufer Q C Araujo, Tara Ellingson, Nicholas U. Schwartz, Connor D. Dietz, Gautam Tammewar, Kaancan Deniz, Manizhe Eslami‐Amirabadi, Andrew G. Breithaupt, Marci Rosenberg, Nhat Minh Bui, Yasmeen Gonzalez, Renaud La Joie, Peter A. Ljubenkov, Katherine L. Possin, Julio C. Rojas, David N. Soleimani‐Meigooni, Yingbing Wang, Adam M. Staffaroni, Melanie L. Stephens, Elena Tsoy, Charles C. Windon, Adam L. Boxer, Gil D. Rabinovici, Bruce L. Miller, Lawren VandeVrede

**Affiliations:** ^1^ Edward and Pearl Fein Memory and Aging Center Weill Institute for Neurosciences Department of Neurology University of California San Francisco San Francisco California US; ^2^ Goizueta Brain Health Institute Emory University Atlanta Georgia US; ^3^ Department of Radiology & Biomedical Imaging Weill Institute for Neurosciences University of California San Francisco San Francisco California US

**Keywords:** Alzheimer's disease, blood‐based biomarkers, neurodegeneration, neurofilament light chain, p‐tau181

## Abstract

**INTRODUCTION:**

Blood‐based biomarkers (BBMs) for Alzheimer's disease (AD), including plasma phosphorylated tau (p‐tau), are increasingly used in clinical practice, but “real‐world” implementation patterns, context‐of‐use (COU), clinical utility, and diagnostic performance are incompletely understood.

**METHODS:**

A retrospective analysis of the first year of BBM use in a tertiary level cognitive neurology subspecialty clinic was conducted, including review of COU, impact on diagnostic certainty, prescription of AD‐related medications, and additional AD‐biomarker testing.

**RESULTS:**

P‐tau181 was ordered frequently to detect AD in a diverse cohort, across a wide spectrum of COU, including typical, early‐onset, and atypical AD presentations; mixed etiology; AD co‐pathology; and borderline symptoms. P‐tau181 impacted diagnostic confidence, AD‐related medication prescription, and follow‐on testing. Renal impairment was a common confounder.

**CONCLUSIONS:**

Implementation of BBMs was feasible and impactful in a memory clinic, especially in a diagnostically complex and diverse patient population, underscoring the need for additional data from real‐world settings.

**Highlights:**

- Blood‐based biomarkers (BBMs) were quickly adopted and accelerated precision diagnosis in a large memory clinic.
- BBM use cases were diverse and included all stages/types of AD and non‐AD syndromes.
- Plasma p‐tau has the potential to impact diagnostic certainty and clinical decision making.
- Diagnostic performance of p‐tau is reasonable, though renal function impacts overall accuracy.

## 1 INTRODUCTION

Several new blood‐based biomarkers (BBMs) of Alzheimer's disease (AD) and related neuropathologies have completed late‐stage validation and are beginning to be used in clinical care, augmenting existing AD biomarkers that test cerebrospinal fluid (CSF) or employ positron emission tomography (PET) to detect the amyloid plaques and tau tangles in AD.^1^ Compared to CSF and PET testing, BBMs offer clear advantages of lower cost and reduced invasiveness, but the precise role of BBM in real‐world settings, especially the specific context‐of‐use (COU) and clinical utility, remains an open question and key priority for the field.^2^, ^3^, ^4^

Tau phosphorylated at threonine 181 (p‐tau181) is a highly specific biomarker for AD and can detect amyloid and tau even in early symptomatic stages of disease,^5^, ^6^, ^7^ though p‐tau217 has subsequently emerged as having improved diagnostic accuracy.^8^, ^9^, ^10^ Recent studies have shown that BBM testing has increased accuracy compared to clinical diagnosis of AD alone, when confirmed against gold‐standard CSF and PET testing, even among specialists and in primary care settings,^11^ leading to calls for incorporation of BBMs into routine clinical practice in any patient with cognitive complaints.^1^ Neurofilament light chain (NfL) is a neuronal cytoskeletal protein that is released from damaged cells into the CSF and blood.^5^, ^12^ NfL can be useful in identifying neurologic injury, and studies have shown that NfL is especially elevated in frontotemporal lobar degeneration (FTLD).^5^, ^13^ Despite elevated levels of NfL being seen in neurodegenerative disease compared to controls on a group level,^5^ the relatively large overlap may limit diagnostic utility on an individual level.

Recently, p‐tau and NfL tests have begun to be implemented in the United States as lab‐developed tests (LDTs) for routine clinical use, though validation data, regulatory approval, and insurance coverage determinations are limited or absent. Additionally, though p‐tau181 and NfL have been studied in research settings and clinical trials and validated in community settings,^10^, ^14^, ^15^ data on clinical impact in routine clinical use in a real‐world clinical settings is incomplete. As BBMs transition from research settings to clinical implementation, experience on BBM implementation by specialists may inform and guide implementation in other settings, including in general neurology and primary care clinics. Therefore, herein we report on clinical use of BBMs in a behavioral neurology clinic in their first year of clinical implementation, highlighting different use cases from patients who had BBM testing performed at the discretion of their clinician as part of their routine diagnostic work‐up.​

## 2 METHODS

A retrospective study was conducted at the Memory and Aging Center (MAC) clinic at the University of California, San Francisco (UCSF), covering the first year of clinical availability of BBMs from July 1 2023 to June 30 2024. The UCSF Institutional Review Board (IRB) approved this study, and informed consent was waived. All clinical encounters (*N* = 1,708) were screened to determine if a clinically available blood test (NfL or p‐tau) was ordered (*N* = 256; 15%). NfL testing was performed by Roche Diagnostics Electrochemiluminescence Immunoassay (ECLIA; LabCorp, Burlington) or Quanterix Single Molecule Array (SiMoA; Mayo Clinic, Rochester). P‐tau tests were performed using Roche Diagnostics ECLIA (p‐tau181) or Fujirebio Lumipulse (p‐tau217; LabCorp, Burlington). Given limited use and availability of p‐tau217 at the time, all subsequent analyses of clinical utility, diagnostic performance, and regression models were restricted to plasma p‐tau181 measured on the Roche Diagnostics ECLIA.

Clinic notes from the day of the order were reviewed by behavioral neurologists blinded to subsequent biomarker result and diagnosis. Each case was clinically staged using all available data, and the pre‐test probability of underlying AD was estimated. Following unblinding of biomarker results, post‐test probability was estimated, and the chart was reviewed for additional biomarker testing or prescription of AD‐related medications. After all data were collected, cases were categorized into specific COU, adapted from appropriate use cases for other modalities.^16^ Amyloid status was determined through positive florbetapir PET scans (*N* = 73), CSF biomarker testing using Roche's ECLIA assay (*N* = 17), or autopsy‐confirmation (*N* = 1). Renal impairment was defined as estimated glomerular filtration rate (eGFR) < 60 mL/min/1.73 m^2^ within 1 year of biomarker test result and was calculated using the Chronic Kidney Disease Epidemiology Collaboration equation without race coefficient as per National Kidney Foundation – American Society of Nephrology. Cases selected to illustrate COU had centiloid quantification utilizing the MIMneuro software.

**RESEARCH IN CONTEXT**

1. **Systematic review**: The authors reviewed the literature using traditional methods, e.g., PubMed. Validated blood‐based biomarkers (BBM) of Alzheimer's disease (AD) are being implemented in clinical settings, but data from real‐world clinical use is limited.
2. **Interpretation**: Herein, the first year of BBM use at a large specialty memory care center is investigated. BBMs were successfully implemented and rapidly adopted, with preliminary evidence of clinical utility and reassuring diagnostic performance, even with first generation BBMs deployed in a diverse and heterogeneous clinical population. BBMs may be a promising tool for providers evaluating cognitive impairment in patients when AD is suspected, though more study is needed before these findings can be generalized to other settings.
3. **Future directions**: Future prospective studies are needed to explore real‐world clinical role of BBMs, especially for additional BBMs, across contexts of use, and in other care settings.

Multivariable logistic regression was used to evaluate the association between p‐tau181 levels and the presence of AD pathology (defined by PET, CSF, or autopsy confirmation), adjusting for clinical impression, estimated glomerular filtration rate (eGFR), Mini‐Mental State Examination (MMSE), age, and sex. Additional models assessed the incremental predictive value of p‐tau181 over clinical diagnosis alone, with model performance evaluated using receiver operating characteristics curve (ROC) curves and DeLong's test for area under the ROC (AUC) comparison. Odds ratios (ORs) and 95% confidence intervals (CIs) were calculated for all predictors. To evaluate the clinical utility of p‐tau181, we examined its impact on three domains: (1) change in clinician‐estimated AD probability, (2) initiation of additional diagnostic testing, and (3) medication initiation or modification. Change in probability was defined as a shift across AD, assessed using Bowker's test. A composite endpoint captured whether a change occurred in any of the three domains and was coded as a binary variable, with patients classified as having experienced a change if they met at least one of the criteria. Nine patients were excluded from the analysis of clinical utility due to missing follow‐up data in one or more of the key domains. Statistical analyses were conducted using R Studio (version 2024.12.1).

## 3 RESULTS

### 3.1 Overall usage after implementation

**FIGURE 1:**
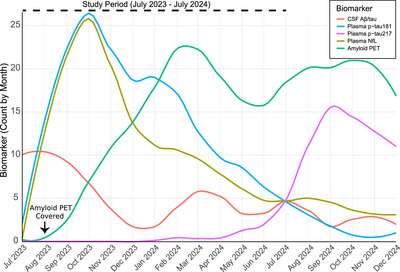
AD biomarker use in a behavioral neurology clinic. Number of AD‐related biomarkers ordered each month, separated by modality (blood, CSF, or PET), over the study period starting after clinical implementation of blood‐based biomarkers in July 2023. Arrow indicates positive coverage determination by CMS for amyloid PET to diagnose AD. AD, Alzheimer's disease; CSF, cerebrospinal fluid; PET, positron emission tomography.

In the first year of clinical implementation, a BBM was ordered in 15% (256/1708) of clinical encounters. NfL was ordered in 181 encounters (146 completed, 12% abnormal), and p‐tau181 was ordered in 228 (175 completed, 67% positive). Figure 1 illustrates the temporal pattern of biomarker ordering across modalities. In October 2023, the Centers for Medicare and Medicaid Services (CMS) announced insurance coverage for amyloid PET imaging to diagnose AD, and following this announcement, a sharp decrease in CSF and BBM usage was observed. Specifically, CSF orders dropped by 67%, and BBM orders by 16%, when comparing the three months before and after the policy change. In contrast, amyloid PET usage increased steadily over the study period, becoming the dominant modality in early 2024. Later, after p‐tau217 was introduced, it gradually supplanted p‐tau181 as the preferred analyte for BBM AD testing, likely due to increased diagnostic accuracy.^8^ NfL testing, which was initially ordered paired with p‐tau181, decreased over the study period, possibly due to the high rate of tests returning in the normal range (88%). Overall, the rate of biomarker testing increased more than three‐fold over the 1‐year study period, following shortly after the United States Food and Drug Administration (FDA) approval of lecanemab in January 2023, which requires biomarker‐confirmation of amyloid prior to treatment initiation.

### 3.2 Clinical characteristics

Plasma p‐tau181 testing was completed in 175 patients during the first year of clinical implementation (Table 1); the average age was 74.1 ± 9.2 years, and 57% were women. Notably, 64% identified as non‐Hispanic White, and 11% were non‐English speakers, representing a more diverse population than research validation studies, even those drawing from community populations.^10^ However, patients who had additional CSF or PET testing were more likely to be non‐Hispanic white (76%, *p* = 0.02; 95% CI_diff_: 2%–18%). Most patients (68%) had a positive p‐tau181 result, though 20% of these (*N* = 35) had evidence of renal impairment, which may falsely elevate p‐tau181 levels.^17^, ^18^ Mean MMSE scores followed an expected gradient across clinical stages: subjective cognitive impairment (SCI; 28.7 ± 1.8), mild cognitive impairment (MCI; 25.6 ± 4.1), and dementia (20.2 ± 6.1). Overall, 92/175 (53%) patients underwent additional testing with amyloid PET (*N* = 73, 79%) or CSF (*N* = 19, 21%) after the plasma biomarker test. Among patients with a positive p‐tau181 result, 83% underwent amyloid PET compared to 53% of those with a negative result. Similarly, 16% of p‐tau181 positive patients had CSF testing, compared to 7% of negative cases. This is consistent with the intended use of the first‐generation p‐tau181 assay as a diagnostic triage tool, where positive results more often prompt additional confirmatory testing.

**TABLE 1:** Patient characteristics by clinical stage, p‐tau result, and context‐of‐use.

		Clinical stage			P‐tau result		Context‐of‐use							
Parameter	Total (*N* = 175)	SCI (*N* = 10)	MCI (*N* = 92)	Dementia (*N* = 73)	Positive (*N* = 119)	Negative (*N* = 56)	Late‐onset amnestic (*N* = 24)	Early‐onset amnestic (*N* = 18)	Atypical AD syndrome^a^ (*N* = 16)	Possible AD copathology^b^ (*N* = 29)	Mixed etiology (Rule out) (*N* = 17)	Mixed etiology (Evaluate) (*N* = 46)	Mixed etiology (Confirm) (*N* = 19)	Borderline symptomatic (*N* = 6)
Age (years), mean ± SD	74.1 ± 9.2	71.1 ± 9.5	73.9 ± 8.3	74.6 ± 10.2	75.3 ± 8.6	71.3 ± 9.9	78.9 ± 5.7	63.6 ± 7.8	73.4 ± 7.1	72.3 ± 11.0	69.5 ± 9.0	76.9 ± 7.1	79.8 ± 5.7	68.8 ± 10.0
**Sex, no. (%)**														
Women	100 (57%)	7 (70%)	49 (53%)	44 (60%)	70 (59%)	30 (54%)	15 (63%)	11 (61%)	10 (63%)	13 (45%)	8 (47%)	27 (59%)	12 (63%)	4 (67%)
Men	75 (43%)	3 (30%)	43 (77%)	29 (40%)	49 (41%)	26 (46%)	9 (38%)	7 (39%)	6 (38%)	16 (55%)	9 (53%)	19 (41%)	7 (37%)	2 (33%)
**Race/Ethnicity, no. (%)**														
Asian	23 (13%)	1 (10%)	10 (11%)	12 (16%)	12 (10%)	11 (20%)	2 (8%)	3 (17%)	1 (6%)	5 (17%)	5 (29%)	5 (11%)	2 (11%)	–
Black/African American	5 (3%)	–	3 (3%)	2 (3%)	4 (3%)	1 (2%)	1 (4%)	–	1 (6%)	–	–	2 (4%)	1 (5%)	–
Hispanic	14 (8%)	–	8 (9%)	6 (8%)	6 (5%)	8 (14%)	2 (8%)	–	–	1 (3%)	1 (6%)	7 (15%)	3 (16%)	–
Non‐Hispanic White	113 (64%)	6 (60%)	63 (68%)	44 (60%)	79 (66%)	34 (61%)	18 (75%)	15 (83%)	12 (75%)	20 (69%)	10 (59%)	24 (52%)	9 (47%)	5 (83%)
Two or more	2 (1%)	1 (10%)	–	1 (1%)	2 (2%)	–	–	–	–	–	1 (6%)	1 (2%)	–	–
Other or unknown	18 (10%)	2 (20%)	8 (9%)	8 (11%)	16 (13%)	2 (4%)	1 (4%)	–	2 (13%)	3 (10%)	–	7 (15%)	4 (21%)	1 (17%)
**Language, no. (%)**														
English	156 (89%)	10 (100%)	85 (92%)	61 (84%)	106 (89%)	50 (89%)	21 (88%)	17 (94%)	16 (100%)	26 (90%)	14 (82%)	38 (83%)	18 (95%)	6 (100%)
Non‐English	19 (11%)	–	7 (8%)	12 (16%)	13 (11%)	6 (11%)	3 (13%)	1 (6%)	–	3 (10%)	3 (18%)	8 (17%)	1 (5%)	–
Positive p‐tau181, no. (%)	119 (68%)	4 (40%)	62 (67%)	53 (73%)	119 (100%)	–	20 (83%)	14 (78%)	13 (81%)	14 (48%)	9 (53%)	34 (74%)	14 (74%)	1 (17%)
**P‐tau181 (pg/mL), mean ± SD**														
Positive	1.88 ± 0.91	1.40 ± 0.26	1.91 ± 1.03	1.88 ± 0.78	1.88 ± 0.91	–	1.81 ± 0.73	2.51 ± 1.00	2.05 ± 0.68	1.85 ± 1.43	1.42 ± 0.27	1.72 ± 0.88	1.92 ± 0.69	1.58 ± 0.0
Negative	0.73 ± 0.15	0.70 ± 0.14	0.73 ± 0.16	0.73 ± 0.15	–	0.73 ± 0.15	0.80 ± 0.14	0.76 ± 0.11	0.73 ± 0.12	0.69 ± 0.14	0.72 ± 0.17	0.70 ± 0.21	0.85 ± 0.07	0.70 ± 0.2
MMSE, mean ± SD	23.6 ± 5.7	28.7 ± 1.8	25.6 ± 4.1	20.1 ± 6.1	22.5 ± 5.9	26.0 ± 4.2	24.9 ± 4.2	20.4 ± 7.6	21.9 ± 6.8	23.1 ± 7.0	26.9 ± 4.1	24.25 ± 4.5	21.4 ± 4.4	28.5 ± 2.3
Renal Impairment, no. (%)	35 (21%)	3 (30%)	15 (16%)	17 (25%)	32 (28%)	3 (6%)	6 (27%)	1 (7%)	–	7 (24%)	3 (19%)	12 (26%)	5 (26%)	1 (17%)
**Additional testing performed, no. (%); %positive**														
Amyloid PET	73 (42%); 71%	4 (40%); 75%	47 (51%); 70%	22 (30%); 73%	58 (49%); 76%	15 (27%); 53%	12 (50%); 92%	10 (56%); 70%	9 (56%); 67%	8 (28%); 38%	10 (59%); 80%	17 (37%); 71%	5 (26%); 100%	2 (33%); 50%
CSF	19 (11%); 63%	1 (10%); 0%	9 (10%); 56%	9 (12%); 78%	11 (9%); 100%	7 (13%); 14%	2 (8%); 50%	2 (11%); 100%	1 (6%); 100%	–	3 (18%); 33%	8 (17%); 63%	2 (11%); 100%	1 (17%); 0%
**Medication prescribed, no. (%)**														
AchE inhibitor	79 (45%)	1 (10%)	39 (42%)	39 (53%)	71 (60%)	8 (14%)	14 (58%)	14 (78%)	9 (56%)	6 (21%)	3 (18%)	23 (50%)	9 (47%)	1 (17%)
Memantine	5 (3%)	–	3 (3%)	2 (3%)	4 (3%)	1 (2%)	–	1 (6%)	–	2 (7%)	1 (6%)	1 (2%)	–	–
Anti‐amyloid antibodies	14 (8%)	–	10 (11%)	4 (5%)	12 (10%)	2 (4%)	4 (17%)	3 (17%)	1 (6%)	–	–	5 (11%)	1 (5%)	–
None	89 (51%)	8 (80%)	49 (53%)	32 (44%)	43 (36%)	46 (82%)	9 (38%)	4 (22%)	6 (38%)	22 (76%)	14 (82)	20 (43%)	9 (47%)	5 (83%)

^a^ Atypical AD syndromes include logopenic variant of primary progressive aphasia (lvPPA, *N* = 9), corticobasal syndrome (CBS, *N* = 4), posterior cortical atrophy (PCA, *N* = 2), and behavioral variant of AD (bvAD, *N* = 1).

^b^ Clinical presentations included behavioral variant of frontotemporal dementia (bvFTD, *N* = 6), vascular dementia (VaD, *N* = 6), Creutzfeldt–Jakob disease (CJD, *N* = 3), dementia with Lewy bodies (DLB, *N* = 3), epilepsy (*N* = 2), traumatic encephalopathy syndrome (TES, *N* = 2), multiple system atrophy (MSA, *N* = 1), non‐fluent variant of PPA (nfvPPA, *N* = 1), progressive supranuclear palsy, Richardson's syndrome (PSP‐RS, *N* = 1), semantic variant of PPA (svPPA, *N* = 1), autism spectrum disorder (ASD, *N *= 1), normal pressure hydrocephalus (NPH, *N* = 1), and medication effect (*N* = 1).

Abbreviations: AD, Alzheimer's disease; MCI, mild cognitive impairment; MMSE, Mini‐Mental State Examination; SCI, subjective cognitive impairment.

### 3.3 Context‐of‐use

**FIGURE 2:**
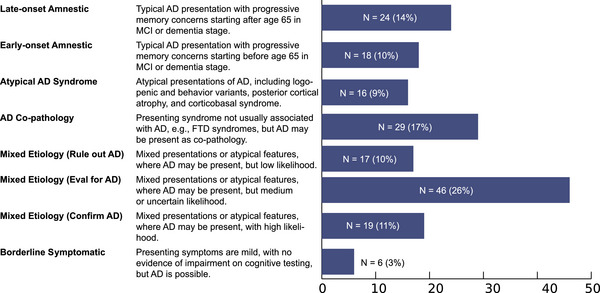
Clinical contexts of use for p‐tau181 testing in a behavioral neurology clinic. Eight identified contexts of use and their relative frequency over 1 year of clinical testing in a real‐world setting.

All p‐tau181 tests were ordered to detect AD, but eight different specific contexts of use were identified (Figure 2). Interestingly, only 14% had typical late‐onset presentations of AD, though this may be partially due to referral and selection bias given the center is a tertiary care referral center with specialization in atypical, early‐onset, and non‐AD neurodegenerative diseases. Indeed, a high proportion of use cases were more diagnostically complex categories, including atypical AD syndromes (9%), mixed etiology (47%), and AD co‐pathology (17%). In particular, the AD co‐pathology group encompassed a wide range of clinical syndromes, including behavioral variant frontotemporal dementia (bvFTD, *N* = 6), vascular dementia (VaD, *N* = 6), Creutzfeldt–Jakob disease (CJD, *N* = 3), dementia with Lewy bodies (DLB, *N* = 3), epilepsy (*N* = 2), among others, supporting the use in non‐AD presentations.^19^ Ten representative cases are presented below as clinical vignettes to highlight different contexts of use.

#### 3.3.1 Late‐onset amnestic

Vignette 1: A 79‐year‐old woman with hyperlipidemia presented with 2 years of progressive memory problems. Her general neurological exam was normal. Neuropsychological testing revealed verbal memory deficit with a score of 25/30 on the MMSE. Brain magnetic resonance imaging (MRI) showed mild precuneus and hippocampal atrophy. She was clinically diagnosed with mild dementia with high suspicion of AD. BBM confirmed AD with elevated p‐tau181 of 2.77 pg/mL (reference < 0.97 pg/mL), whereas NfL was normal at 5.12 pg/ml (reference < 7.64 pg/ml). A subsequent [18F]florbetapir PET scan for eligibility for amyloid‐targeted treatment was visually diffusely positive with a centiloid (CL) of 75. The patient was approved after board review, and amyloid‐targeted treatment was initiated.

Vignette 2: An 87‐year‐old man with a history of hypertension and obstructive sleep apnea (OSA) initially presented to clinic a decade prior with 2 years of progressive memory difficulties (MMSE 28/30). MRI demonstrated mild cerebrovascular disease burden and global atrophy, for which he was given a diagnosis of MCI. Over the following years, his level of impairment initially remained stable, but then declined in the setting of life stressors, and the patient was started on acetylcholinesterase (AchE) inhibitors due to clinical concern for AD. These medications were poorly tolerated, and after BBM testing revealed a negative p‐tau181 (0.69 pg/mL) and normal NfL, they were discontinued.

#### 3.3.2 Early‐onset amnestic

Vignette 3: A 58‐year‐old woman with hypertension, fibromyalgia, chronic fatigue syndrome, irritable bowel syndrome, post‐traumatic stress disorder, and anxiety presented for evaluation of “brain fog.” Neurological exam suggested a component of functional overlay. Neuropsychological testing found deficits in learning, memory, attention, and processing speed. Brain MRI noted mild asymmetric atrophy. She was diagnosed with MCI. BBMs were sent to evaluate for AD, though clinical suspicion was intermediate. P‐tau181 was positive (1.47 pg/mL) and NfL was normal. The elevated p‐tau181 result prompted amyloid PET, which was visually focally positive (21 CL), suggestive of early stage of disease. The patient considered amyloid‐targeted treatments but ultimately opted for lifestyle intervention and active surveillance.

#### 3.3.3 Atypical AD syndrome

Vignette 4: A 67‐year‐old man, with history of hyperlipidemia and remote concussion, presented with a 2‐year history of progressive visuospatial difficulties, trouble with navigation, reading, writing, performing calculations, telling left from right, and controlling the left side of his body. Neurological exam was notable for saccade apraxia, left‐sided neglect, mild left‐sided rigidity, limb ideomotor apraxia, hyperreflexia, and gait apraxia. MRI was notable for prominent dorsoparietal atrophy. The patient was diagnosed with corticobasal syndrome (CBS). P‐tau181 testing was negative (0.67 pg/mL), whereas NfL was elevated to 9.13 pg/mL (reference < 4.61), suggesting CBS was due to a 4R‐tauopathy, in particular corticobasal degeneration (CBD).

Vignette 5: A 78‐year‐old man with history of atrial flutter and late‐onset epilepsy presented for progressive changes in memory, visuospatial, and language abilities following a seizure 3 years prior. Neurological exam was notable for soft and dysarthric voice, prominent apraxia, and slow effortful right‐sided rapid alternating movements with dystonic posturing of his right hand. Neuropsychological testing revealed difficulties in executive functioning, verbal learning, language, and visuoconstruction. The patient was diagnosed with MCI and CBS. P‐tau181 was elevated (1.28 pg/mL) and NfL was normal (3.84 pg/mL, reference < 7.64), resulting in a diagnosis of CBS due to AD.^20^

#### 3.3.4 AD co‐pathology

Vignette 6: An 83‐year‐old man with remote focal seizures, sensorineural hearing loss, hypertension, and coronary artery disease presented with memory complaints, but was noted to have a dysexecutive profile on neurocognitive testing. Other pertinent findings included bouts of fluctuating alertness, anosmia, and rapid eye movement (REM) behavior disorder. His neurological exam showed parkinsonism with cogwheeling, decreased arm swing, and stooped posture. He was diagnosed with DLB, but given his age, primary amnestic complaints, and posterior‐predominant atrophy on MRI, AD was thought to be contributing. P‐tau181 was positive (1.69 pg/mL) and AD was subsequently confirmed with visually positive amyloid PET (CL 48). A DAT scan showed diminished tracer uptake, suggesting presynaptic striatal dopaminergic deficit, and the final diagnosis was DLB due to α‐synuclein with AD copathology.

#### 3.3.5 Mixed etiology: Rule out AD

Vignette 7: A 74‐year‐old man with a history of hyperlipidemia, bipolar disorder, and hypothyroidism presented to clinic a decade prior with 2 years of mild memory and word‐finding deficits. Initial MRI revealed scattered subcortical white matter disease, and he was diagnosed with mild cognitive impairment secondary to vascular disease and untreated depression. Over the intervening years, he had worsening word‐finding and mildly increased parietal atrophy. Given the patient's age, progressive concerns, and mild atrophy, the clinician opted to query for possible AD, though suspicion was low. BBM testing revealed a negative p‐tau181 (0.85 pg/mL), with subsequent amyloid PET also visually negative (CL ‐4), ruling out AD as a contributor.

#### 3.3.6 Mixed etiology: Evaluate for AD

Vignette 8: A 65‐year‐old man with atrial fibrillation (not on anticoagulation), hypertension, and hyperlipidemia presented with progressive word finding difficulties following transient sudden‐onset language difficulty 2 years prior. Neurological exam showed trouble with word retrieval, spatial memory, executive changes, and subtle episodic memory deficits. Neuropsychological testing revealed prominent executive dysfunction affecting verbal memory encoding with relative preservation of consolidation; MMSE 28/30. Brain MRI demonstrated a chronic cortical infarct in the left parietal lobe and multiple smaller chronic infarcts in the same vascular territory, suggesting an embolic event. He was diagnosed with MCI due to vascular disease, but comorbid AD was thought to be possibly contributing, so p‐tau181 testing was ordered. P‐tau181 was on the threshold at 0.97 pg/mL (reference: < 0.98 pg/mL), but the borderline result coupled with moderate suspicion resulted in an amyloid PET being ordered, which was visually focally positive (CL 1). The patient was considered for amyloid‐targeted treatment, but mitigation of stroke risk was considered the priority.

#### 3.3.7 Mixed etiology: Confirm AD

Vignette 9: An 87‐year‐old woman with a history of diabetes, hypertension, hyperlipidemia, and hypothyroidism presented with 1 year of cognitive symptoms (MMSE 15/28), and a recent brain MRI notable for moderate hippocampal atrophy, white matter disease, and lobar microhemorrhage. The patient was diagnosed with mild dementia with high suspicion for AD given age and amnestic presentation. P‐tau181 was normal (0.49 pg/mL) and NfL was mildly elevated (58.6 pg/mL, reference < 51.2 pg/mL), suggesting non‐AD neurodegenerative disease, later confirmed with a visually negative amyloid PET (CL ‐34). Ultimately, further work‐up led to a multifactorial diagnosis, including vascular disease, limbic‐predominant age‐related TDP‐43 encephalopathy (LATE), and cerebral amyloid angiopathy (CAA).

#### 3.3.8 Borderline symptoms

Vignette 10: A 69‐year‐old woman with sleep problems, anxiety, and positive family history of AD was followed in clinic over 7 years for continued cognitive concerns. Her initial workup included negative AD biomarkers in CSF. Neurologic exam was normal, and neuropsychological testing was above average, though high educational attainment limited test utility. Brain MRI was normal for age. She was diagnosed with SCI with low suspicion of a neurodegenerative disease. However, due to continued cognitive concerns, p‐tau testing was obtained twice over 2 years, with both results within the normal range (0.59 pg/mL, 0.49 pg/mL). Of note, the patient was counseled ahead of testing that because of low predicted prevalence in her cohort and low clinical suspicion, testing would only be useful to rule out AD. However, negative testing provided reassurance to the patient that cognitive symptoms were unlikely AD, and OSA was found as potential explanation for new cognitive symptoms.

### 3.4 Clinical utility

**FIGURE 3:**
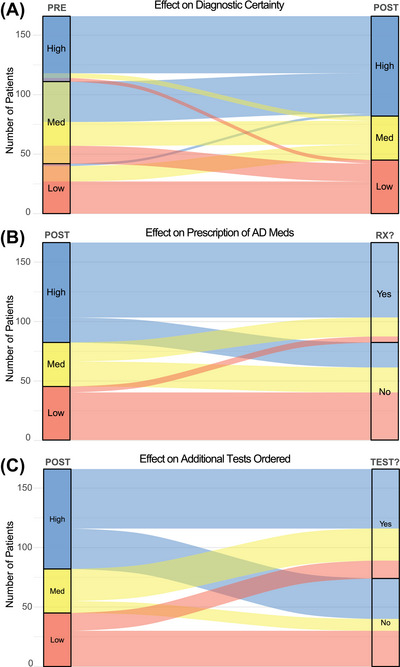
Retrospective clinical utility of p‐tau181 testing in a behavioral neurology clinic. (A) Estimated pre‐ and post‐test diagnostic certainty of p‐tau181 for the presence of AD neuropathology. (B) Impact on prescription of an AD‐related medication by post‐test diagnostic certainty. (C) Impact of additional CSF or PET testing by post‐test diagnostic certainty. AD, Alzheimer's disease; CSF, cerebrospinal fluid.

To assess the clinical utility of p‐tau181 testing, we examined whether results led to changes in (1) estimated diagnostic certainty; (2) prescription of medications typically used to treat AD, including acetylcholinesterase inhibitors (AchE), memantine, and/or newer amyloid‐targeted treatments; and (3) orders for additional confirmatory testing, including PET and CSF. Pre‐ to post‐test, most cases remained stable in prediction of likelihood of AD neuropathology (Figure 3A), but 71 of 166 patients (43%) shifted categories, with a reduction in the percentage of clinically indeterminate (medium) cases from 42% (*N* = 69) to 22% (*N* = 37). Bowker's test of symmetry confirmed that this distributional change was statistically significant (*χ* ^2^ = 24.0, *p *< 0.001), reflecting an overall increase in diagnostic certainty. Unsurprisingly, patients with high post‐test probability of AD were more likely to receive pharmacologic treatments associated with AD (*N* = 62, 73%, Figure 3B) when compared to patients with medium (*N* = 17, 43%) or low suspicion (*N* = 5, 11%). Prescriptions in patients with low suspicion for AD may be partially explained by the use of AchE inhibitors in synucleinopathies, e.g., DLB. Finally, the highest percentage of patients referred for additional testing were those with medium certainty post‐test certainty (*N* = 26, 65%, Figure 3C), compared to those with high (*N* = 49, 58%) or low (*N* = 14, 30%) certainty, though additional testing in the high certainty group is partially due to the need for CSF or PET confirmation for amyloid‐targeted treatment eligibility.

### 3.5 Diagnostic performance

**FIGURE 4:**
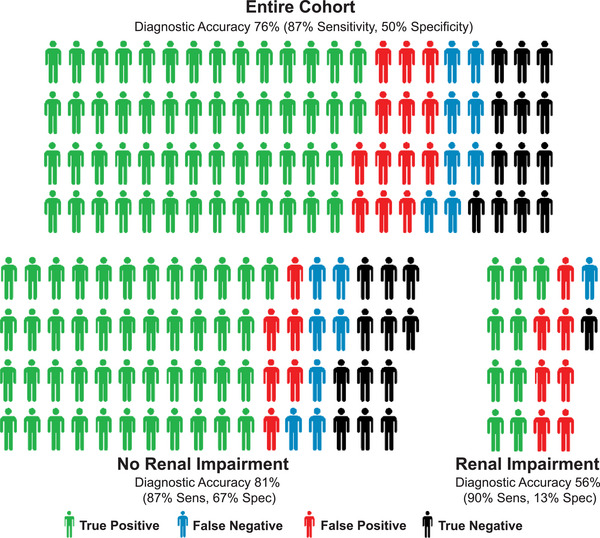
Diagnostic performance of p‐tau181 testing in a behavioral neurology clinic. Color‐coded by diagnostic result after CSF or PET testing for the entire cohort (top) and within patients with and without renal impairment (bottom). Diagnostic performance should be interpreted cautiously given inherent selection bias, as only 53% of patients who had BBM testing had additional testing with CSF or PET ordered (*N* = 88 completed), often due to continued diagnostic uncertainty. BBM, blood‐based biomarker; CSF, cerebrospinal fluid; PET, positron emission tomography.

To evaluate the diagnostic accuracy of p‐tau181 for detecting AD, we compared test results against reference standards (CSF, amyloid PET, or autopsy). Among the full sample that received reference standard assessment (*N* = 88), p‐tau181 achieved an overall diagnostic accuracy of 76%, with high sensitivity (87%) but limited specificity (50%) (Figure 4, top panel). Performance varied substantially by renal function. Among patients without evidence of renal impairment, accuracy improved to 81%, with similar sensitivity (87%) and a higher specificity (67%). In contrast, in patients with renal impairment, accuracy was only 56%. These results are consistent with prior studies showing that renal dysfunction may elevate plasma p‐tau181 levels independent of AD pathology, leading to false‐positive results.^17^, ^18^ To further evaluate test performance while adjusting for clinical and demographic factors, we constructed a series of multivariable logistic regression models using confirmed AD pathology as the outcome. In models adjusting for clinical impression, MMSE, renal function (eGFR), age, and sex, p‐tau181 remained the strongest independent predictor of AD (OR = 4.48, 95%CI: 1.54–17.19, *p* = 0.016). Other predictors, including MMSE and pre‐test clinical impression, were not independently associated with AD in the fully adjusted model. Model discrimination improved with the inclusion of p‐tau181 (AUC = 0.72 vs. 0.60 for clinical impression alone), and further increased to 0.80 when age, sex, and eGFR were added. These findings support the diagnostic utility of p‐tau181 to augment clinical judgment, while highlighting the importance of renal function as a modifying factor in biomarker interpretation.

## 4 DISCUSSION

This study provides a comprehensive real‐world evaluation of blood‐based testing and broader biomarker use in a tertiary memory clinic in the first year of clinical use, finding that blood biomarkers can be rapidly implemented and widely applied, including in cohorts and contexts of use not included in typical research validation efforts, with potential impact on diagnostic certainty, prescription of AD‐directed medication, and need for additional testing. Importantly, given the diverse population, unanticipated contexts of use, and confounds like renal impairment, additional prospective studies in real‐world settings are urgently needed, especially as these tools are increasingly implemented in non‐specialist settings. Importantly, the specific BBMs used in this study were first generation LDTs and lacked FDA review and CMS coverage determination and yet were ordered in 15% of patient encounters in a behavioral neurology clinic. We suspect that clarity regarding regulatory approval, intended COU, and insurance coverage will increase clinical implementation and use, and, since the conclusion of our study, the FDA has cleared the first blood‐based assay to aid in the diagnosis of AD (Fujirebio Lumipulse plasma p‐tau217/Ab42), marking a major milestone for regulatory‐approved biomarker use in clinical practice.

Notably, our clinic‐based cohort was more racially and ethnically diverse than research studies in similar disease cohorts at our center,^19^ with 36% of patients self‐identifying as non‐white and 11% non‐primary English speakers, more closely reflecting racial and ethnic compositions of the local community.^21^ However, additional confirmatory biomarker testing with PET and CSF remained more common in white patients, suggesting clinical implementation of BBMs may increase access to biomarker testing and reduce barriers to AD diagnosis for underrepresented populations in a memory clinic.^22^ Additionally, this study highlights the need for inclusivity in pre‐implementation validation efforts, which may be critical due to potential differences in biomarker performance between cohorts, though these disparities may be explained by social determinants.^23^, ^24^

BBM were frequently ordered in diagnostically complex presentations, including early‐onset, atypical, and mixed presentations, an enriched population in a tertiary referral center. Surprisingly, only 14% of patients were categorized as typical late‐onset amnestic presentations, whereas nearly half were considered to have mixed etiology, highlighting the need for studies using real‐world data that extend beyond curated research cohorts. Importantly, BBM results influenced clinical decision‐making across these cohorts. A significant proportion of patients (25%) had shifts in clinician‐estimated AD probability, affecting the prescription of AD‐directed medications and/or need for additional confirmatory diagnostic testing. These findings reinforce that BBMs are not only diagnostically informative but clinically actionable.

Regarding diagnostic performance, p‐tau181 demonstrated high sensitivity and moderate specificity for diagnosing AD. However, these data should be interpreted cautiously given a clear selection bias: patients routed for more testing may be more likely to have results that conflict with clinical suspicion, warranting additional investigation. Notably, patients with positive p‐tau181 results were significantly more likely to undergo confirmatory testing than those with negative results (60% vs. 37%, OR = 2.5, *p* = 0.007), suggesting that cases with expected and concordant findings—particularly at the extremes of clinical probability—may have been underrepresented. This likely enriched the reference‐tested subgroup for diagnostically ambiguous cases and may have contributed to the observed reduction in specificity. Diagnostic accuracy in the real world would be best assessed in a cohort where every patient had confirmatory testing, and diagnostic performance using this approach is reassuringly comparable to research validation efforts.^10^, ^15^ However, as seen in other studies, diagnostic performance was reduced among individual with renal impairment, a common comorbidity (21%) in the population as a whole. These findings underscore the importance of incorporating renal function and other confounders into BBM interpretation workflows and recommendations.

Our study has limitations, especially the use of first generation LDT and p‐tau181, an analyte with slightly worse diagnostic performance than p‐tau217.^8^ However, plasma p‐tau217 testing was only recently implemented in our clinic, limiting our ability to study longitudinal changes in diagnostic certainty and clinical care. Additionally, because the clinical immunoassays used did not provide paired measurements of the corresponding non‐phosphorylated or total tau peptides, we were unable to compute p‐tau/total‐tau ratios, which have been previously shown to mitigate the impact of chronic kidney disease (CKD) on p‐tau increases.^25^ We also did not systematically ascertain cardiovascular risk factors, which recent studies indicate may attenuate the strength of association between plasma p‐tau and AD neuropathology.^26^, ^27^ Future investigations would benefit from inclusion of p‐tau217 (and other analytes) across multiple platforms, especially as diagnostic performance is dependent on these factors.^28^ The retrospective design limits the ability to definitively understand clinical utility and impact, and future studies could prospectively collect data pre‐ and post‐test, following similar studies that evaluated the impact of amyloid PET on clinical management.^29^ Lastly, our study setting was a large behavioral neurology clinic that is a tertiary referral center for atypical AD and FTLD presentations, so clinical utility is expected to differ from general neurology or primary care settings, and additional studies in these settings are warranted.

Overall, our findings provide insight into clinical implementation, patterns of use, test performance, and clinical impact across a wide spectrum of neurodegenerative syndromes, complementing recent studies conducted in primary and secondary care settings.^11^ Specifically, our results highlight both the promise and challenges of implementing BBMs in routine care. Although BBMs offer a scalable and accessible tool with high potential for clinical impact across the spectrum of neurodegenerative disease, optimal use will depend on context‐specific interpretation, especially in atypical cases and among patients with comorbidities such as renal impairment. Future work should aim to refine BBM appropriate use recommendations and further integrate findings obtained from real‐world clinical data to guide best practices for implementation.^30^

## Consent Statement

The UCSF Institutional Review Board (IRB) approved this study, and informed consent was waived for all human subjects.

## CONFLICT OF INTEREST STATEMENT

The authors declare no conflicts of interest. Author disclosures are available in the supporting information.

## DISCLOSURES

Dr. Schwartz has received funding from 2T32AG023481 and P30AG062422 and has served as an advisor for Topline Bio. Dr. Breithaupt reported grant funding supported by the American Brain Foundation, Eisai, and the Alzheimer's Association. Dr. La Joie reported grants from the NIH, the Alzheimer's Association, the US Department of Defense as well as personal fees from GE Healthcare outside the submitted work. Dr. Possin receives research support from the NIH, the Global Brain Health Institute, the Alzheimer's Association, and the Rainwater Charitable Foundation. Dr. Rojas reported serving as site principal investigator for clinical trials sponsored by Eli Lilly, Eisai, and Amylyx. Dr. Soleimani‐Meigooni reported grants from the NIH and serves on an Alzheimer's Association research council. Dr. Staffaroni reported grants from the NIH, the Bluefield Project to Cure Frontotemporal Dementia, and the Association for Frontotemporal Degeneration; personal fees from Alector, Prevail Therapeutics/Eli Lilly, Passage Bio, Takeda, and the Alzheimer's Drug Discovery Foundation; and licensing fees from Datacubed Health. Dr. Tsoy reported grants from the NIH, the Alzheimer's Association, and the Global Brain Health Institute. Dr. Windon reports grants from the Alzheimer's Association and NIH, and has received honoraria from LCN, Kinetix Group, and Onviv. Dr. Boxer reported grants from the NIH, the Rainwater Charitable Foundation, the GHR Foundation, and the Bluefield Project and personal fees from Alector, Arvinas, Alchemab, Alexion, Amylyx, Arkuda, Arrowhead, Eli Lilly, Muna, Neurocrine, Ono, Oscotec, Pfizer, Switch, Transposon, and Unlearn AI. Dr. Rabinovici reported grants from National Institutes of Health; consulting fees from C2N, Eli Lilly, Alector, Merck, Roche, and Novo Nordisk; data safety monitoring board fees from Johnson & Johnson; and grants from Avid Radiopharmaceuticals, GE Healthcare, Life Molecular Imaging, and Genentech. Dr. Miller reported serving on the scientific advisory board of the Bluefield Project to Cure Frontotemporal Dementia; the John Douglas French Alzheimer's Foundation; Fundación Centro de Investigación Enfermedades Neurológicas, Madrid, Spain; Genworth; the Kissick Family Foundation; the Larry L. Hillblom Foundation; and the Tau Consortium of the Rainwater Charitable Foundation; serving as a scientific advisor for the Arizona Alzheimer's Consortium; Massachusetts General Hospital Alzheimer's Disease Research Center; and the Stanford University Alzheimer's Disease Research Center; receiving royalties from Cambridge University Press, Elsevier, Guilford Publications, Johns Hopkins Press, Oxford University Press, and the Taylor & Francis Group; and receiving grants for the University of California San Francisco Frontotemporal Dementia Core, from the Bluefield Project to Cure Frontotemporal Dementia, and from the National Institute on Aging for the US–South American Initiative for Genetic‐Neural‐Behavioral Interactions in Human Neurodegenerative Diseases. Dr. VandeVrede reported grants from the NIH, the Alzheimer's Association, and Shenandoah Foundation, serving as the site principal investigator for clinical trials sponsored by Merck and Biogen, and consulting fees from Biogen, Roche, and Siemens. No other disclosures were reported.

## Supporting information



Supporting Information
